# Putative Activation of the CB1 Cannabinoid Receptors Prevents Anxiety-Like Behavior, Oxidative Stress, and GABA Decrease in the Brain of Zebrafish Submitted to Acute Restraint Stress

**DOI:** 10.3389/fnbeh.2020.598812

**Published:** 2021-01-18

**Authors:** Waldo Lucas Luz, Mateus Santos-Silva, Patrick Bruno Cardoso, Nadyme Assad, Edinaldo Rogério da Silva Moraes, Alan Barroso Araújo Grisólia, Danielle Valente Braga, Luana Ketlen Reis Leão, Suellen Alessandra Soares de Moraes, Adelaide da Conceição Passos, Evander de Jesus Oliveira Batista, Amauri Gouveia, Karen R. H. Matos Oliveira, Anderson Manoel Herculano

**Affiliations:** ^1^Laboratory of Experimental Neuropharmacology, Institute of Biological Sciences, Federal University of Pará, Belém, Brazil; ^2^Laboratory of Protozoology, Tropical Medicine Center, Federal University of Pará, Belém, Brazil; ^3^Laboratory of Neuroscience and Behavior, Federal University of Pará, Belém, Brazil

**Keywords:** anxiety, acute stress, CB1 receptors, oxidative stress, GABA, zebrafish

## Abstract

Anxiety disorder is a well-recognized condition observed in subjects submitted to acute stress. Although the brain mechanisms underlying this disorder remain unclear, the available evidence indicates that oxidative stress and GABAergic dysfunction mediate the generation of stress-induced anxiety. Cannabinoids are known to be efficient modulators of behavior, given that the activation of the cannabinoid receptors type-1 (CB1 receptors) induces anxiolytic-like effects in animal models. In the present study, we aimed to describe the effects of the stimulation of the CB1 receptors on anxiety-like behavior, oxidative stress, and the GABA content of the brains of zebrafish submitted to acute restraint stress (ARS). The animals submitted to the ARS protocol presented evident anxiety-like behavior with increased lipid peroxidation in the brain tissue. The evaluation of the levels of GABA in the zebrafish telencephalon presented decreased levels of GABA in the ARS group in comparison with the control. Treatment with ACEA, a specific CB1 receptor agonist, prevented ARS-induced anxiety-like behavior and oxidative stress in the zebrafish brain. ACEA treatment also prevented a decrease in GABA in the telencephalon of the animals submitted to the ARS protocol. Overall, these preclinical data strongly suggest that the CB1 receptors represent a potential target for the development of the treatment of anxiety disorders elicited by acute stress.

## Introduction

Anxiety disorder is a global public health problem (Alonso et al., 2018). The World Health Organization (WHO) estimates that approximately one-third of the world’s population develops anxiety at some time, with many of the cases being triggered by traumatic events (Bandelow and Michaelis, 2015). Acute stress is an important source of anxiety disorder in humans and several studies have correlated this event with imbalances in the brain neurotransmitter systems such as the serotoninergic, glutamatergic, and GABAergic systems (Gemikonakli et al., 2019; Assad et al., 2020; de Abreu et al., 2020a; Feder et al., 2020). There is good evidence that the pharmacological modulation of the GABAergic system can prevent the anxiety-like behavior induced by acute and chronic stress (Nuss, 2015; Assad et al., 2020; Farajdokht et al., 2020). Pro-oxidant events in the brain tissue are also known to be closely associated with anxiety disorder elicited by post-traumatic events (Sousa et al., 2018). Based on these findings, it is reasonable to assume that compounds able to modulate GABAergic synapses and inhibit oxidative stress will have protective effects against anxiety disorder induced by acute stress.

The endocannabinoid system has emerged as an important regulator of behavior in humans and animal models (Volkow et al., 2016; Papagianni and Stevenson, 2019). The physiological activity of the endocannabinoid system is mediated through the activation of two classes of proteins, called type 1 (CB1) and type 2 (CB2) cannabinoid receptors (Kendall and Yudowski, 2016). While the CB2 receptors are located mostly in the peripheral organs, in particular in the cells involved in the immune response, the CB1 receptors are concentrated primarily in the Central Nervous System, the CNS (Kendall and Yudowski, 2016). The CB1 receptors are expressed most in the olfactory bulb, the lateral striatum, cerebellum, and striatal nuclei, and are also present in the brain regions that control fear and anxiety, such as the amygdala, hippocampus, and dorsal telencephalon (Lam et al., 2006; Imperatore et al., 2015). The association between the CB1 receptors and anxiety-like behavior is controversial, given that low concentrations of CB1 agonists have been shown to produce anxiolytic-like behaviors, whereas higher doses induced an anxiogenic effect (Rey et al., 2012; Stewart and Kalueff, 2014). The effects of CB1 stimulation on oxidative injuries also remain unclear, although treatment with AEA has been shown to reduce the production of Reactive Oxygen Species (ROS) and protect hippocampal neurons against oxidative injury (Lipina and Hundal, 2016; Gallelli et al., 2018). Our research group has recently demonstrated that Acute Restraint Stress (ARS) induces anxiety-like behavior, associated with GABAergic dysfunction in the zebrafish brain (Assad et al., 2020), and based on these findings; the present study evaluated the preventive effects of treatment with the CB1 agonist on the neurochemistry of anxiety-like behavior induced by ARS in zebrafish.

## Materials and Methods

### Drugs and Reagents

Arachidonyl-2′-chloroethylamide hydrate (ACEA, 97% purity CAS: 220556-69-4) was purchased from Sigma Aldrich—São Paulo. Reagents for quantification of GABA by HPLC (sodium acetate, homoserine, methanol, and gamma-aminobutyric acid), lipid peroxidation (*N*-methylphenylindole, methanesulfonic acid, and malondialdehyde), and for non-protein thiol assay [5,5′-Dithiobis(2-nitrobenzoic acid), N-acetylcysteine] were purchased from Sigma Aldrich (Brazil) and were from analytical grade.

### Animals and Housing

A total of 156 adult-long-fin zebrafish (*Danio rerio*) of both sexes (50:50 ratio) were purchased from a local distributor, in Ananindeua, Pará (Brazil). These animals were kept for at least one week in 50 L tanks (50 cm × 35 cm × 30 cm) under appropriate conditions, as described by Assad et al. (2020), that is, at 27 ± 2°C, pH = 6.5, and a 14/10 h light/dark cycle, with a density of one animal per liter, fed twice a day. The tanks were maintained under constant mechanical and chemical filtration.

### Ethics Statement

The presentstudy was approved by the Ethics Committee on Research in Experimental Animals of the Federal University of Pará (CEPAE—UFPA: 213-14). All procedures were conducted following the guidelines of the Brazilian National Council for the Control of Animal Experimentation (CONCEA).

### Acute Stress Protocol and Behavioral Test

Zebrafish were cold-anesthetized and injected intra-abdominally with either saline solution (SAL, 0.9%) or 1 mg/Kg ACEA dissolved in 0.005% DMSO. Drug dilution and treatments were performed 30 min before the behavioral experiments to assure drug distribution and reach in zebrafish brain as performed by de Carvalho et al. (2019) and Assad et al. (2020). The fish were then transferred to individual plastic aquaria, in which they were kept for 30 min. The animals assigned to the stress group were submitted to ARS as described by Dal Santo et al. (2014). For this, the fish were inserted into 1.5 ml microtubes with openings at both ends for 90 min.

The animals were then submitted to the Novel Tank Diving Test, using a protocol modified from those described by Egan et al. (2009) and Cachat et al. (2010). The exploration of the apparatus by the animal was recorded for 10 min using a digital camera (Cyber-shot DSC-W710 BR4), with the following parameters being quantified in the X-Plo Rat software: time at the top, squares crossed, erratic swimming, and freezing. The swimming trajectory of the fish was evaluated using ZebraTrack software, following the approach of Pinheiro-da-Silva et al. (2017). The behavioral tests were conducted between 7 AM and 6 PM. Following the test, each animal was cold-anesthetized and its brain was dissected in cold PBS buffer solution for the posterior biochemical assays.

### High-Performance Liquid Chromatography (HPLC)

The GABA content in the brains of the control and ACEA groups was determined using a Shimadzu (LC-10 AD) HPLC with an injection circuit attached to an LC-20AT pump, analytical Shimadzu C18 column, and fluorescence detector (RF-10AXL). The mobile phase (phase A) consisted of a solution containing 50 mM of sodium acetate, 5% methanol, and 2-propanol. Phase B contained 70% methanol. Immediately after the behavior task, the animals were cryoanesthetized individually and their brains were dissected as soon as possible. The samples were then transferred to culture dishes containing Na^+^—Hanks buffer (128 mM NaCl; 4 mM KCl; 1 mM MgCl_2_; 2 mM CaCl_2_, 12 mM glucose, and 20 mM HEPES, pH. 7.2) and maintained for 20 min in a CO_2_ stove at 37°C. The analysis matrix was prepared by adding 1% TCA, followed by centrifugation for 10 min at 5,000 rpm. The matrix was made by mixing 60 μl of each sample in 10 μl of methanolic o-phthaldialdehyde and pH 9.5 borate buffer. The final volume of the samples was vortexed and injected into the system for analysis after 5 min. The GABA levels in each sample were determined using a standard GABA curve, with the values being expressed as a percentage of the control (telencephalon = 0.14 ± 0.002 μM/mg ptn and mesencephalon = 0.02 ± 0.006 μM/mg ptn). The protein content was determined by the Bradford method.

### Non-protein Thiol Assay (NPSH)

The current protocol was adapted from that of Marcon et al. (2018). The brains were stored in Tris-HCl buffer, and the samples were first defrosted and sonicated before analysis. An equal volume of 6% trichloroacetic acid was added to each sample, which was later centrifuged at 3,000 rpm for 10 min at 4°C. The supernatant was collected and added to 1 M TFK buffer (26 mM KH_2_PO_4_, 25 mM K_2_HPO_4_, pH 7.2) to react for 15 min with 10 mM 5, 5-dithio-bis-2-nitrobenzoic acid (DTNB) at a proportion of 2:1. The subsequent analysis of non-protein thiol was based on the standard curve of N-acetylcysteine, measured by its absorbance at a wavelength of 406 nm. The protein levels were determined using the Bradford method, with the values being expressed as a percentage of the control (7.4 ± 0.5 μmol/mg ptn).

### Lipid Peroxidation Assay

The brains of the control and treated zebrafish were dissociated mechanically in pH 7.4 Tris-HCl buffer (1 M Tris base, 1 M chloride acid) and centrifuged at 5600 rpm for 10 min at 4°C. The product of lipid peroxidation was determined in the supernatant using 20 mM of pH 7.4 Tris-HCl, 10.3 mM of N-methyl-2phenylindole (NMFI), and methanesulfonic acid at 45°C. The lipid peroxidation was analyzed based on the standard curve of the malondialdehyde (MDA) concentrations, measured by its absorbance at a wavelength of 570 nm (de Carvalho et al., 2019). The MDA concentration was quantified in nmols per milligram of protein determined using the Bradford method. The values were expressed as a percentage of the control (8.1 ± 1.6 nmol/mg ptn).

### Statistical Analysis

The statistical analyses considered *n* = 18 subjects per group in the behavioral tests and *n* = 12 subjects per group in the biochemical assays. The normal distribution of the data was determined by the Shapiro-Wilk test. The means were compared between pairs of groups using Student’s *t*, and when more than two groups were involved; a two-way analysis of variance (ANOVA) was applied (Factor 1: treatment or not, Factor 2: stressed or not) followed by the Bonferroni *post hoc* test. All the analyses were run in the BioEstat 5.0 software, with a *p* < 0.05 significance level being considered in all cases.

## Results

**Figure 1:**
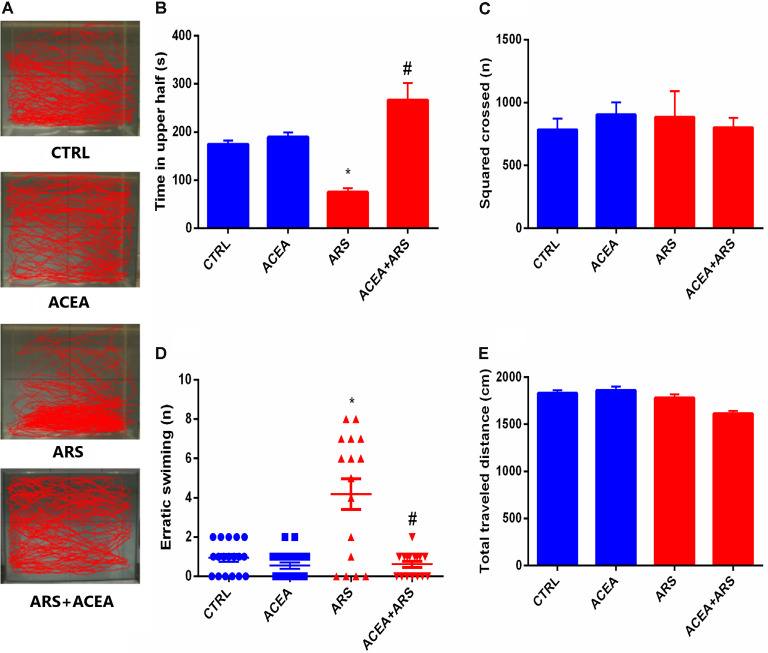
Effect of CB1 agonist on the locomotion **(A)** and behavior parameters of zebrafish submitted to Acute Restraint Stress (ARS): time spent on top in seconds **(B)** score crossed **(C)** erratic swimmer **(D)** and total traveled distance **(E)**. Values showed as MEAN ± SEM (ANOVA one way, Bonferroni *post hoc* test. *Compared to control; ^#^compared to ARS *p* < 0.05).

To verify the effect of the ARS protocol on the anxiety-like behavior of the study fish, the control group and ACEA-treated animals were submitted to the Novel Tank Diving Test as described in the methods. The trajectories of the two groups in the apparatus are shown in Figure 1A which, together with the data presented in Figure 1B indicates that zebrafish submitted to ARS protocol spent less time on the top of the apparatus when compared with the control group. This anxiogenic effect was prevented by ACEA treatment which has increased the exploration of zebrafish on the top of apparatus in animals submitted to ARS when compared with non-treated animals exposed to acute stress. These findings were confirmed by the evaluation of erratic swimming, which was fully prevented by ACEA (Figure 1D). We also observed that neither the ARS nor the ACEA treatment affected zebrafish motor activity, as demonstrated by the square-crossing and total distance traveled data (Figures 1C,E).

**Figure 2:**
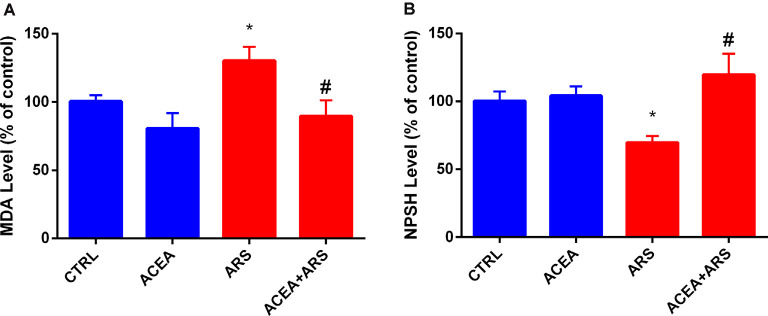
Effect of CB1 agonist in the lipid peroxidation **(A)** and non-protein thiol content **(B)** in the brain of zebrafish submitted to ARS. Values showed as a percent of control (ANOVA one way, Bonferroni *post hoc* test. *Compared to control; ^#^compared to ARS *p* < 0.05).

The effects of ARS on zebrafish brain was confirmed by the biochemical analysis, which showed that the animals submitted to ARS presented over-production of MDA levels (about 30%) in brains of animals submitted to the ARS protocol (Figure 2A). This pro-oxidant effect was followed by a reduction of 20% of sulfhydryl groups in their brains in comparison with the control group (Figure 2B). Our data also described that MDA levels in brains of zebrafish submitted to acute stress and treated with ACEA were similar to the control group, as shown in Figure 2A. We also observed that ACEA treatment prevented depletion of sulfhydryl groups in zebrafish brains submitted to ARS as described in Figure 2B.

**Figure 3:**
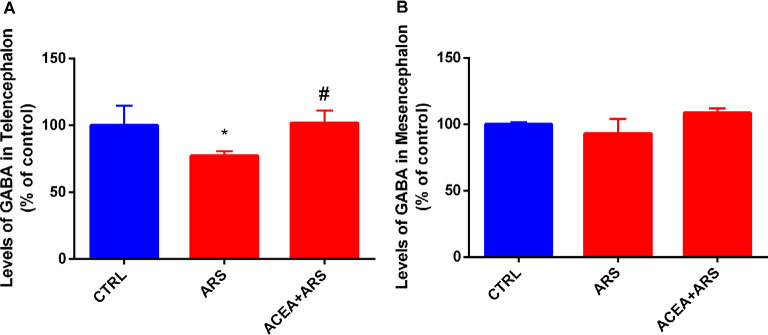
GABA levels in the telencephalon **(A)** and mesencephalon **(B)** of zebrafish submitted to ARS in the presence or absence of CB1 agonist. Values showed as a percent of control (ANOVA one way, Bonferroni *post hoc* test. *Compared to control; ^#^compared to ARS *p* < 0, 05).

The neurochemical evaluation of the GABA content showed that ARS decreases GABA values by about in 23% in the telencephalic region of the zebrafish exposed to the ARS protocol. We demonstrated that treatment with ACEA was able to prevent the decrease in the GABA induced by ARS in the telencephalic region of the zebrafish brain (Figure 3A). Our data also evidenced that ARS does not alter GABA levels in the zebrafish mesencephalon in comparison with non-stressed animals (Figure 3B).

## Discussion

In the present study, we demonstrated that the stimulation of the CB1 receptors prevents anxiety-like behavior and the neurochemical alterations induced by ARS in zebrafish. The zebrafish is widely known to be an excellent model animal for the evaluation of alterations in the CNS induced by stressful conditions (Stewart et al., 2012; Caramillo et al., 2015; de Abreu et al., 2020b). The data presented in the current study are consistent with the literature, given that the zebrafish submitted to ARS swam erratically and spent less time at the top of the apparatus which is recognized as altered behavior. As shown previously, this modified activity is consistent with anxiogenic-like behavior (Piato et al., 2011). Our findings are consistent with the latter study, which demonstrated that acute restraint stress induces anxiety-like behavior without altering the locomotion of the zebrafish, and expand this understanding by showing that treatment with CB1 receptor agonist prevents the anxiety-like behavior induced by acute restraint stress. Data from Tran et al. (2016) are in agreement with our study since it was evidenced that treatment with antagonists of CB1 receptors induces anxiety-like behavior in zebrafish. The understanding of the influence of CB1 agonists on anxiety is still controversial; given that both anxiogenic and anxiolytic effects have been reported. Our findings nevertheless support the hypothesis that activation of the CB1 receptors by ACEA prevents the anxiety induced by acute restraint stress. It is also important to clarify that although ACEA represents a high specific CB1 agonist (Hillard et al., 1999; Ma et al., 2015; Simone et al., 2015; Yang et al., 2020), posterior studies using other specific agonists and antagonists are essential to confirm that CB1 receptors control the biochemical and behavioral alterations elicited by acute restraint stress.

Data presented in the current study are also supported by histological evidence showing that CB1 receptors are expressed in telencephalic regions of zebrafish brain (Lam et al., 2006; Watson et al., 2008) and as previously evidenced, the telencephalon represents a brain structure closely involved in the control of anxiety-like behavior in zebrafish (Maximino et al., 2013). In current work, the protective effects exerted by treatment with CB1 agonist against the deleterious impacts of acute stress on the zebrafish brain were confirmed by the biochemical evidence. As we have shown here, acute restraint stress favored the pro-oxidant status of the zebrafish brain. This is clear from the significant reduction in the levels of the sulfhydryl compounds and the greater lipid peroxidation in the brains of the animals submitted to acute stress. These findings are consistent with the previous studies that have shown that the over-production of ROS is a mediating phenomenon in stress-induced anxiety disorder (Marcon et al., 2018). Although the precise molecular mechanism involved in cannabinoid regulation of ROS production remains unclear, some studies already describe that activation of CB1 receptors can inhibit oxidative stress by a mechanism mediated by activation of peroxisome proliferator-activated receptors (PPARs). Data described by Palomba et al. (2015) showed that CB1 stimulation by ACEA promotes PPARs activation and prevents oxidative stress in hypothalamic cells. In this same study, the involvement of the ACEA-CB1-PPARs pathway to prevent oxidative stress was ratified by data showing that knockdown of CB1 receptors completely abolishes the effect of ACEA on the PPARs activation. Based on these pieces of evidence, posterior studies need to be performed to demonstrate the possible participation of PPARs in the antioxidant and anxiolytic effect exerted by CB1 activation in zebrafish submitted to acute restraint stress.

The neurochemical analysis also demonstrated alterations in a specific region of the zebrafish brain following acute restraint stress. While the GABA content of the mesencephalon was not altered, the levels of this neurotransmitter decreased significantly in the telencephalic region of the stressed zebrafish. Several studies have described the telencephalon of the zebrafish as a region homologous to the mammalian limbic system, which controls anxiety in humans (von Trotha et al., 2014). In fact, previous studies indicate the close association of the zebrafish telencephalon with the control of anxiety-like behavior (Assad et al., 2020). The putative activation of the CB1 receptors also prevented a decrease in the GABA content of the telencephalon of stressed zebrafish. Although the association between the CB1 receptors and both oxidative stress and the GABAergic system is not fully understood, our data have suggested that CB1 stimulation maintained the redox status, prevent oxidative stress, and ensure the homeostasis of the GABAergic synapse. Taken together, the present preclinical study gives strong evidence that CB1 receptors could be a potential target for the treatment of anxiety disorder generated by posttraumatic events.

## Data Availability Statement

The raw data supporting the conclusions of this article will be made available by the authors, without undue reservation.

## Ethics Statement

The animal study was reviewed and approved by the Ethics Committee on Research in Experimental Animals of the Federal University of Pará (CEPAE—UFPA: 213-14).

## Author Contributions

WL, MS-S, and AH: conceived and designed the experiments. WL, PC, NA, EM, and DB: performed the experiments. KO, ABG, AG, and LL: analyzed the data. EB, SM, and AP: contributed reagents, materials, and analysis tools. AH, EB, and KO: wrote the article. All authors contributed to the article and approved the submitted version.

## Conflict of Interest

The authors declare that the research was conducted in the absence of any commercial or financial relationships that could be construed as a potential conflict of interest.
